# Tracking Architecture Based on Dual-Filter with State Feedback and Its Application in Ultra-Tight GPS/INS Integration

**DOI:** 10.3390/s16050627

**Published:** 2016-05-02

**Authors:** Xi Zhang, Lingjuan Miao, Haijun Shao

**Affiliations:** School of Automation, Beijing Institute of Technology (BIT), Beijing 100081, China; miaolingjuan@bit.edu.cn (L.M.); shaohaijun5@126.com (H.S.)

**Keywords:** baseband signal preprocessing, dual-filter, state feedback, ultra-tight GPS/INS integration, navigation filter with expanded dimension

## Abstract

If a Kalman Filter (KF) is applied to Global Positioning System (GPS) baseband signal preprocessing, the estimates of signal phase and frequency can have low variance, even in highly dynamic situations. This paper presents a novel preprocessing scheme based on a dual-filter structure. Compared with the traditional model utilizing a single KF, this structure avoids carrier tracking being subjected to code tracking errors. Meanwhile, as the loop filters are completely removed, state feedback values are adopted to generate local carrier and code. Although local carrier frequency has a wide fluctuation, the accuracy of Doppler shift estimation is improved. In the ultra-tight GPS/Inertial Navigation System (INS) integration, the carrier frequency derived from the external navigation information is not viewed as the local carrier frequency directly. That facilitates retaining the design principle of state feedback. However, under harsh conditions, the GPS outputs may still bear large errors which can destroy the estimation of INS errors. Thus, an innovative integrated navigation filter is constructed by modeling the non-negligible errors in the estimated Doppler shifts, to ensure INS is properly calibrated. Finally, field test and semi-physical simulation based on telemetered missile trajectory validate the effectiveness of methods proposed in this paper.

## 1. Introduction

The navigation errors of GPS are not divergent with time, but a receiver usually performs less well in highly dynamic situations because there are external signals to be dealt with. On the other hand, though INS has the problem of error accumulation, it realizes autonomous navigation and is robust to vehicle dynamics. Since GPS and INS have complementary advantages, their integration can overcome each other's defects and provide a better navigation performance [1]. Unlike loose or tight GPS/INS integration, in which GPS is just an aid to INS, the ultra-tight GPS/INS integration uses INS to couple with GPS tracking channels. In this sense, the ultra-tight form is especially applicable to high dynamic situations [2], and has gradually become the mainstream approach for designing GPS/INS integrated systems.

The ultra-tight GPS/INS integration originates from vector tracking [3]. Its core concept is using the known ephemeris and the corrected inertial navigation information to calculate tracking parameters. Namely, satellite signal locking is assisted by the integrated navigation filter, and GPS tracking channels no longer remain independent of each other. According to the structural design of the filter models, the ultra-tight GPS/INS integration methods can be roughly divided into two types. In one case, a single high-dimensional filter is used to estimate the GPS tracking parameters of each channel and navigation parameters together [4,5], and in the other case, each channel has a baseband signal pre-filter which estimates the tracking parameters for itself, and the integrated navigation filter just needs to estimate the navigation parameters. The second category is actually a form of federated filtering, and usually has higher computational efficiency [6,7,8].

The baseband signal preprocessing realizes optimal estimation of signal characteristic quantities by KF. Thus, under the application background of the ultra-tight GPS/INS integration, it can restrain phase noise more effectively than the loop filter of the classical GPS tracking channel [9,10]. For each channel, the baseband signal preprocessing is generally completed by a single pre-filter [11]. Both carrier tracking errors and code tracking errors are included in the state vector, namely, these two kinds of tracking errors are tightly coupled through the state and observation equations. However, the precision of carrier and code loops do not have the same order of magnitude. The code loop has great tolerance for tracking errors due to the long wavelength of CA code, while the carrier loop is much more sensitive [12,13]. As preprocessing continues, the code tracking errors would be delivered to the carrier loop and cause the degradation of carrier tracking precision or even loss of lock. In addition, even if the baseband signal preprocessing is applied in a GPS receiver, the Doppler shifts estimated under highly dynamic conditions may still contain significant errors. In the ultra-tight GPS/INS integration, these incorrect GPS outputs eventually lead the estimation of INS errors to be contaminated [14]. That generally means the whole system suffers performance degradation, or even completely crashes.

To solve these problems, this paper makes creative contributions in both the tracking and navigation domain. Two independent pre-filters with state feedback are constructed to replace the classical loop filters. The advantages of decoupling between carrier tracking and code tracking are analyzed in detail. Since the single-filter and dual-filter preprocessing models are constructed based on the same principle, their comparison can be free from the influence of other factors. In fact, compared with a popular error-state pre-filter, even the single-filter model has better tracking performance due to the benefits of state feedback. In the ultra-tight GPS/INS integration, local carrier generation is still controlled by state feedback, and the carrier frequency derived from the external navigation information is just used to correct the state feedback value. Meanwhile, a specific integrated navigation filter is presented by taking the pseudorange rate estimation error in each channel as the additional state variable. This scheme ensures the estimation of original state variables, especially the INS errors, is immune to these GPS tracking errors, and hence enables the integrated system to maintain high accuracy even when GPS receiver operates under harsh conditions.

The rest of this paper is organized as follows: first, a baseband signal preprocessing model is designed based on a dual-filter structure, while the state feedback control values of carrier and code loops are derived in detail. Then, the specific integrated navigation filter is proposed, and a concrete approach to aid the tracking channel is elaborated. Furthermore, the results of field tests and semi-physical simulation are given and discussed. Finally, the work in this paper is summarized.

## 2. Construction of Baseband Signal Preprocessing Model Based on Dual-filter Structure

The classical GPS tracking channel, which typically adopts second-order or third-order phase control technology, has fixed loop bandwidth. Although a narrower bandwidth is helpful to reduce the thermal noise, in highly dynamic situations, the tracking channel may not retain useful high frequency information and suffers from significant dynamic stress error. These usually lead to signal distortion and tracking loss. Thus, a trade-off between noise resistance and response to dynamics is required while setting the loop bandwidth. In view of the abovementioned fact, the classical control approach cannot cope well with high dynamics, and hence KF, which belongs to the modern control category, is taken into account. KF is a kind of optimal estimator, which allows precise modeling of the tracking loop. With its help, baseband signal preprocessing can achieve accurate control of the local signal generation, even in highly dynamic situations [15].

**Figure 1:**
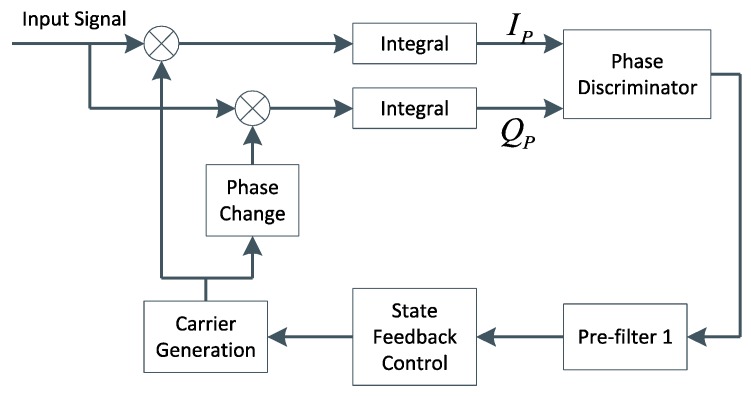
The carrier loop using pre-filter.

The baseband signal preprocessing model constructed in this paper contains two pre-filters. In this model, the estimation of carrier and code tracking errors are relatively separate, the same as the manner adopted by classical GPS receiver. Figure 1 just takes carrier loop for example to show the details of this model. It is observed that the loop filter in the classical tracking channel has been replaced by a pre-filter equipped with state feedback. Obviously, designing a suitable KF is the key to accurate carrier tracking.

This paper sets $X_{k}^{carr}={\left[\Delta \theta_{k}^{carr}\;f_{k}^{carr}\;{\dot{f}}_{k}^{carr}\right]}^{T}$ as the state vector of pre-filter 1, where k is the epoch, $\Delta \theta_{k}^{carr}$ is the carrier phase error (unit: rad), $f_{k}^{carr}$ is the input carrier frequency, and ${\dot{f}}_{k}^{carr}$ is the changing rate of the input carrier frequency. These state variables have the following recurrence relations:

(1) $$\begin{array}{c} \Delta \theta_{k,k-1}^{carr}=\frac{1}{T_{coh}}\int_{0}^{T_{coh}}(\Delta \theta_{k-1}^{carr}+2\pi f_{k-1}^{carr}t+\pi {\dot{f}}_{k-1}^{carr}t^{2})dt-\frac{1}{T_{coh}}\int_{0}^{T_{coh}}2\pi u_{k-1}^{carr}tdt\\ =\Delta \theta_{k-1}^{carr}+\pi f_{k-1}^{carr}T_{coh}+\frac{1}{3}\pi {\dot{f}}_{k-1}^{carr}T_{coh}^{2}-\pi u_{k-1}^{carr}T_{coh} \end{array}$$

where $T_{coh}$ is the coherent integration time, $u_{k-1}^{carr}$ is the local carrier frequency at epoch k−1.

The output of carrier phase discriminator at epoch *k*, notated as $\Delta \theta_{k,k-1}^{carr}$, is the average carrier phase error over an integration interval, due to the presence of Doppler shift. Thus, this output can be expanded as:

(2) $$\begin{array}{c} \Delta \theta_{k,k-1}^{carr}=\frac{1}{T_{coh}}\int_{0}^{T_{coh}}(\Delta \theta_{k-1}^{carr}+2\pi f_{k-1}^{carr}t+\pi {\dot{f}}_{k-1}^{carr}t^{2})dt-\frac{1}{T_{coh}}\int_{0}^{T_{coh}}2\pi u_{k-1}^{carr}tdt\\ =\Delta \theta_{k-1}^{carr}+\pi f_{k-1}^{carr}T_{coh}+\frac{1}{3}\pi {\dot{f}}_{k-1}^{carr}T_{coh}^{2}-\pi u_{k-1}^{carr}T_{coh} \end{array}$$

Unfortunately, if Equation (2) is directly used to generate the observation model for pre-filter 1, there will be a time lag between the state vector and observation [16]. Hence, by moving $\Delta \theta_{k-1}^{carr}$, $f_{k-1}^{carr}$ and ${\dot{f}}_{k-1}^{carr}$ to the left side of the equal sign, Equation (1) is rewritten as follows (more details are available in Appendix A):

(3) $$\left\{ \begin{array}{l} \Delta \theta_{k-1}^{carr}=\Delta \theta_{k}^{carr}-2\pi f_{k}^{carr}T_{coh}+\pi {\dot{f}}_{k}^{carr}T_{coh}^{2}+2\pi u_{k-1}^{carr}T_{coh}\\ f_{k-1}^{carr}=f_{k}^{carr}-{\dot{f}}_{k}^{carr}T_{coh}\\ {\dot{f}}_{k-1}^{carr}={\dot{f}}_{k}^{carr} \end{array}\right.$$

Substitution of Equation (3) into Equation (2) results in:

(4) $$\Delta \theta_{k,k-1}^{carr}=\Delta \theta_{k}^{carr}-\pi f_{k}^{carr}T_{coh}+\frac{1}{3}\pi {\dot{f}}_{k}^{carr}T_{coh}^{2}+\pi u_{k-1}^{carr}T_{coh}$$

Then, in terms of Equations (1) and (4), the state equation and observation equation of pre-filter 1 can be expressed as:

(5) $$\left\{ \begin{array}{l} X_{k}^{carr}=\Phi_{k,k-1}^{carr}X_{k-1}^{carr}+M_{k-1}^{carr}+W_{k-1}^{carr}\\ Z_{k}^{carr}=H_{k}^{carr}X_{k}^{carr}+N_{k}^{carr}+V_{k}^{carr} \end{array}\right.$$

where:

(6) $$\Phi_{k,k-1}^{carr}=\left[\begin{array}{ccc} 1 & 2\pi T_{coh} & \pi T_{coh}^{2}\\ 0 & 1 & T_{coh}\\ 0 & 0 & 1 \end{array}\right]$$

(7) $$M_{k-1}^{carr}={\left[\begin{array}{ccc} -2\pi T_{coh}u_{k-1}^{carr} & 0 & 0 \end{array}\right]}^{T}$$

(8) $$H_{k}^{carr}=\left[\begin{array}{ccc} 1 & -\pi T_{coh} & \frac{1}{3}\pi T_{coh}^{2} \end{array}\right]$$

(9) $$N_{k}^{carr}=\pi T_{coh}u_{k-1}^{carr}$$

$Z_{k}^{carr}$ is the observation, and can be directly obtained from carrier phase discriminator, namely:

(10) $$Z_{k}^{carr}=\Delta \theta_{k,k-1}^{carr}=\arctan\frac{Q_{p}}{I_{p}}$$

$W_{k-1}^{carr}$, $V_{k}^{carr}$ represent the system noise and observation noise, respectively. Assume these two noises have the following characteristics:

(11) $$\left\{ \begin{array}{l} E\left[W_{k}\right]=0,E\left[W_{k}W_{j}^{T}\right]=Q_{k}^{carr}\delta_{kj}\\ E\left[V_{k}\right]=0,E\left[V_{k}V_{j}^{T}\right]=R_{k}^{carr}\delta_{kj}\\ E\left[W_{k}V_{j}^{T}\right]=0 \end{array}\right.$$

where $\delta_{kj}$ is the Kronecker delta. Then, the corresponding iterative algorithm for pre-filter 1 is given below:

(12) $${\hat{X}}_{k,k-1}^{carr}=\Phi_{k,k-1}^{carr}{\hat{X}}_{k-1}^{carr}+{\hat{M}}_{k-1}^{carr}$$

(13) $$P_{k,k-1}^{carr}=\Phi_{k,k-1}^{carr}P_{k-1}^{carr}\Phi_{k,k-1}^{carr,T}+Q_{k-1}^{carr}$$

(14) $$K_{k}^{carr}=P_{k,k-1}^{carr}H_{k}^{carr,T}{\left(H_{k}^{carr}P_{k,k-1}^{carr}H_{k}^{carr,T}+R_{k}^{carr}\right)}^{-1}$$

(15) $$P_{k}^{carr}=\left(I-K_{k}^{carr}H_{k}^{carr}\right)P_{k,k-1}^{carr}$$

(16) $${\hat{X}}_{k}^{carr}={\hat{X}}_{k,k-1}^{carr}+K_{k}^{carr}\left(Z_{k}^{carr}-N_{k}^{carr}-H_{k}^{carr}{\hat{X}}_{k,k-1}^{carr}\right)$$

The carrier loop is in essence a Phase Lock Loop (PLL), and aims to achieve the elimination of the carrier phase error. Since $\Delta \theta_{k+1}^{carr}=\Delta \theta_{k}^{carr}+2\pi \left(f_{k}^{carr}-u_{k}^{carr}\right)T_{coh}+\pi {\dot{f}}_{k}^{carr}T_{coh}^{2}$, by assuming $\Delta \theta_{k+1}^{carr}=0$, the feedback at epoch *k* can be obtained as:

(17) $$u_{k}^{carr}=\frac{\Delta {\hat{\theta }}_{k}^{carr}}{2\pi T_{coh}}+{\hat{f}}_{k}^{carr}+\frac{1}{2}{\hat{\dot{f}}}_{k}^{carr}T_{coh}$$

It can be seen that $u_{k}^{carr}$ is not exactly equivalent to the estimated input carrier frequency ${\hat{f}}_{k}^{carr}$. And further discussions about $u_{k}^{carr}$ and ${\hat{f}}_{k}^{carr}$ can be found in Section 3 and Section 4.1.

Similarly, the state vector of pre-filter 2 is set to $X_{k}^{c\text{ode}}={\left[\Delta \theta_{k}^{code}\;f_{k}^{code}\;{\dot{f}}_{k}^{code}\right]}^{T}$, where $\Delta \theta_{k}^{code}\;$is the code phase error (unit: chip), $f_{k}^{code}$ is the input code frequency, and ${\dot{f}}_{k}^{code}$ is the changing rate of the input code frequency. Meanwhile, in consideration of the following formula:

(18) $$f_{d}=f^{carr}-f_{IF}=\frac{f_{L1}}{f_{CA}}(f^{code}-f_{CA})$$

where $f_{d}$ is the Doppler shift, $f_{IF}$ is the Intermediate Frequency (IF), $f_{L1}$ (1575.42 MHz) is the L1 carrier frequency, $f_{CA}$ (1.023 MHz) is the nominal code frequency, the state equation and observation equation of pre-filter 2 can be written as:

(19) $$\left\{ \begin{array}{l} X_{k}^{code}=\Phi_{k,k-1}^{code}X_{k-1}^{code}+M_{k-1}^{code}+W_{k-1}^{code}\\ Z_{k}^{code}=H_{k}^{code}X_{k}^{code}+N_{k}^{code}+V_{k}^{code} \end{array}\right.$$

where:

(20) $$\Phi_{k,k-1}^{code}=\left[\begin{array}{ccc} 1 & T_{coh} & \frac{1}{2}T_{coh}^{2}\\ 0 & 1 & T_{coh}\\ 0 & 0 & 1 \end{array}\right]$$

(21) $$M_{k-1}^{code}={\left[\begin{array}{ccc} -T_{coh}u_{k-1}^{code} & 0 & 0 \end{array}\right]}^{T}$$

(22) $$H_{k}^{code}=\left[\begin{array}{ccc} 1 & -\frac{1}{2}T_{coh} & \frac{1}{6}T_{coh}^{2}\\ 0 & \frac{f_{L1}}{f_{CA}} & 0 \end{array}\right]$$

(23) $$N_{k}^{code}={\left[\begin{array}{cc} \frac{1}{2}T_{coh}u_{k-1}^{code} & 0 \end{array}\right]}^{T}$$

(24) $$Z_{k}^{code}=\left[\begin{array}{c} \Delta \theta_{k,k-1}^{code}\\ f_{k}^{carr}-f_{IF}+f_{L1} \end{array}\right]$$

$W_{k-1}^{c\text{ode}}$, $V_{k}^{c\text{ode}}$ are respectively the system and observation noises of pre-filter 2, while the observation $\Delta \theta_{k,k-1}^{code}$ obtained from code phase discriminator can be denoted by:

(25) $$\Delta \theta_{k,k-1}^{code}=\frac{1}{2}\frac{\sqrt{I_{E}^{2}+Q_{E}^{2}}-\sqrt{I_{L}^{2}+Q_{L}^{2}}}{\sqrt{I_{E}^{2}+Q_{E}^{2}}+\sqrt{I_{L}^{2}+Q_{L}^{2}}}$$

$u_{k-1}^{code}$ is the local code frequency at epoch k−1. Moreover, at epoch k, the local code frequency, namely the feedback for code tracking, can be expressed as:

(26) $$u_{k}^{code}=\frac{\Delta {\hat{\theta }}_{k}^{code}}{T_{coh}}+{\hat{f}}_{k}^{code}+\frac{1}{2}{\hat{\dot{f}}}_{k}^{code}T_{coh}$$

Since code estimation is processed after carrier stripping, its errors have no effect on carrier tracking. At the same time, once these two pre-filters are skillfully tuned, they can accurately track GPS signals in high dynamic situations. Taking pre-filter 1 for example, its equivalent bandwidth can be calculated from the elements of the gain matrix $K_{k}^{carr}$ and is proportional to $Q_{k}^{carr}\left(1,1\right)$, $Q_{k}^{carr}\left(2,2\right)$ and $Q_{k}^{carr}\left(3,3\right)$ [17]. Herein, $Q_{k}^{carr}\left(1,1\right)$, $Q_{k}^{carr}\left(2,2\right)$ and $Q_{k}^{carr}\left(3,3\right)$ are the diagonal elements of $Q_{k}^{carr}$. $Q^{carr}\left(1,1\right)$ and $Q^{carr}\left(2,2\right)$ (the epoch k is omitted to reveal these two values are constant) can be obtained from the receiver clock model [11], while $Q_{k}^{carr}\left(3,3\right)$ is related to the vehicle dynamics. If $Q_{k}^{carr}\left(3,3\right)$ is estimated in real-time by any efficient adaptive algorithm, the loop bandwidth will vary in a time-varying optimal manner. This paper uses another more practical method: $Q^{carr}\left(3,3\right)$ is set to a large constant value based on the general knowledge of vehicle dynamics. Since $Q^{carr}\left(1,1\right)$ and $Q^{carr}\left(2,2\right)$ are unchanged, the low variances of $\Delta {\hat{\theta }}_{k}^{carr}$ and ${\hat{f}}_{k}^{carr}$ can be maintained [3]. In other words, although the variance of ${\hat{\dot{f}}}^{carr}$ increases, phase and frequency estimations which normally deserve concern can hardly be affected. Additionally, $R^{carr}$ is set according to the knowledge of the phase noise variance, while $P_{0}^{carr}$ is usually larger than $Q^{carr}$ to leave space for state corrections. In this case, pre-filter 1 gradually settles down to a wide bandwidth, and hence is robust to high dynamics. Benefiting from the above mentioned advantages, this baseband signal preprocessing model can be appropriate for highly dynamic situations and make GPS have better tracking and navigation performance.

## 3. Realization of the Specific Ultra-tight GPS/INS Integration

The carrier tracking loop employing pre-filter 1 is in essence equivalent to a third-order loop. However, a *N-th* order phase locked loop cannot correctly track the signal whose phase varies by time to the Nth or higher-order power (the estimates of signal parameters can still have low variance, but not low bias) [18]. That indicates, even improved by baseband signal processing, an independent GPS receiver is vulnerable to extreme high dynamics, and may give inaccurate tracking and navigation results.

**Figure 2:**
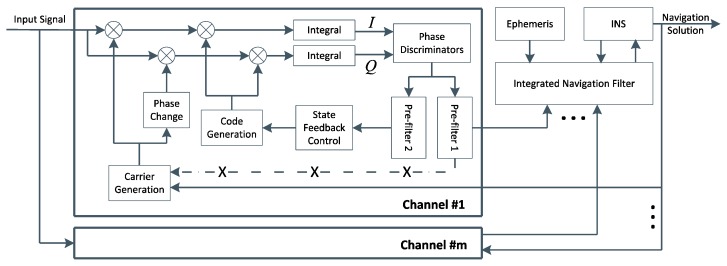
The structure of the ultra-tight GPS/INS integration.

On the contrary, INS is robust to vehicle dynamics. Based on this fact, INS can be used to assist GPS, which usually means baseband signal processing is connected closely with the estimation of navigation parameters. In the ultra-tight GPS/INS integration shown in Figure 2, the integrated navigation filter is responsible for obtaining corrected inertial navigation information, and delivering them back to GPS tracking channels. Because the feedback from the corrected inertial navigation information has the advantage of high accuracy in a short time, GPS tracking channel can perform better under highly dynamic conditions. The detailed work procedure of this ultra-tight GPS/INS integration is as follows: in each GPS tracking channel, in-phase and quadrature signals are processed by phase discriminators and pre-filters to estimate tracking parameters, such as carrier frequency and code phase; the observations of the integrated navigation filter can be converted from tracking parameters as well as deduced from inertial navigation information and satellite ephemeris, and then the corrected inertial navigation information can be obtained; finally, auxiliary tracking parameters, carrier frequency, for example, can be deduced from these external navigation information and satellite ephemeris, and then be applied to assist signal tracking. Through the above analysis, it can be seen that identifying conversion relations between tracking parameters and navigation parameters as well as finding a rational way to assist tracking are the keys to designing a good ultra-tight GPS/INS integration.

In the ideal situation, pseudorange rate and variation of pseudorange output by GPS tracking channel can be respectively derived from:

(27) $${\dot{\rho }}_{G}=-f_{d}\frac{c}{f_{L1}}$$

(28) $$\Delta \rho_{G}=\Delta \theta^{code}\frac{c}{f_{CA}}$$

where c is the speed of light. But in fact, under highly dynamic conditions, the carrier phase of satellite signal may be proportional to time to the power of N or higher value, and hence the estimated carrier frequency may contain a large error $\delta f^{carr}$. Since the incorrect GPS outputs could destroy the estimation of INS errors, it is necessary to analyze this error in detail. Therefore Equation (27) is rewritten as:

(29) $$\begin{array}{c} {\dot{\rho }}_{G}=-\left({\hat{f}}^{carr}-f_{IF}\right)\frac{c}{f_{L1}}\\ =-\left(f_{d}+\delta f^{carr}\right)\frac{c}{f_{L1}} \end{array}$$

On the other hand, Equation (28) can remain unchanged due to the fact that the code loop dynamic stress error is usually small. $\rho_{G}$, which represents the current pseudorange output by the GPS tracking channel, can be calculated from $\Delta \rho_{G}$ and the previous pseudorange. Then, in Earth Centered Earth Fixed (ECEF) coordinate system, the observation equation of integrated navigation filter can be expressed as:

(30) $$\left\{ \begin{array}{l} \delta \rho =\rho_{I}-\rho_{G}=e_{1}\delta x+e_{2}\delta y+e_{3}\delta z-\delta t_{u}+v_{\rho }\\ \delta \dot{\rho }={\dot{\rho }}_{I}-{\dot{\rho }}_{G}=e_{1}\delta \dot{x}+e_{2}\delta \dot{y}+e_{3}\delta \dot{z}-\delta t_{ru}+\delta v_{G}+v_{\dot{\rho }} \end{array}\right.$$

where $\rho_{I}$, ${\dot{\rho }}_{I}$ respectively represent the pseudorange and pseudorange rate deduced from inertial navigation information and satellite ephemeris, δx, δy, δz, $\delta \dot{x}$, $\delta \dot{y}$, $\delta \dot{z}$ are vehicle position errors and velocity errors in ECEF coordinate system, $e_{1}$, $e_{2}$, $e_{3}$ are unit vectors in the x, y, and z direction, $\delta t_{u}$, $\delta t_{ru}$ represent clock bias and drift respectively, $\delta v_{G}$ is the error of pseudorange rate output by the GPS tracking channel and equals to $\delta f^{carr}c/f_{L1}$, $v_{\rho }$, $v_{\dot{\rho }}$ are observation noises. It is noted that Equation (30) should usually be transformed into the geodetic coordinate system to facilitate INS error correction. Then, taking all the available tracking channels into consideration, the observation matrix $\tilde{H}$ can be obtained naturally, where the superscript "~" denotes the navigation filter designed in this paper.

If there are m satellites being locked by tracking channels, the state vector of the integrated navigation filter can be expressed as:

(31) $$\tilde{X}={\left[\begin{array}{ccccc} X & \delta v_{G,1} & \delta v_{G,2} & \cdots & \delta v_{G,m} \end{array}\right]}^{T}$$

where X is the transpose of the state vector adopted by a traditional integrated navigation filter, namely

(32) $$X=\left[\varphi_{E}\;\varphi_{N}\;\varphi_{U}\;\delta v_{E}\;\delta v_{N}\;\delta v_{U}\;\delta L\;\delta \lambda \;\delta h\;\varepsilon_{bx}\;\varepsilon_{by}\;\varepsilon_{bz}\;\nabla_{bx}\;\nabla_{by}\;\nabla_{bz}\;\delta t_{u}\;\delta t_{ru}\right]$$

and the first 15 variables of X represent three INS error states each in attitude, velocity, position, gyro bias, accelerometer bias. Accordingly, the state transition matrix Φ and the noise matrix G of the traditional integrated navigation filter can be expanded to:

(33) $$\tilde{\Phi }=\left[\begin{array}{cc} \Phi_{17\times 17} & 0_{17\times m}\\ 0_{m\times 17} & I_{m\times m} \end{array}\right]$$

(34) $$\tilde{G}=\left[\begin{array}{cc} G_{17\times 8} & 0_{17\times m}\\ 0_{m\times 8} & I_{m\times m} \end{array}\right]$$

In addition, $\tilde{H}$ can also be written as:

(35) $$\tilde{H}=\left[\begin{array}{cc} H_{2m\times 17} & \left[\begin{array}{c} 0_{m\times m}\\ I_{m\times m} \end{array}\right] \end{array}\right]$$

where H is the traditional observation matrix. The specific information of Φ, G and H can be easily found in many references [19,20,21,22] and will not be discussed in detail here.

According to the conversion relations indicated by Equations (27) and (28), the corrected inertial navigation information can in turn feed back into GPS tracking channels. The corresponding carrier frequency estimate is usually accurate enough, and can be used to directly control local carrier generation. However, in the new baseband signal preprocessing model, it is hoped that the phase error can return to 0 in the shortest amount of time, so the external information assists in local carrier generation through correcting the state feedback value, and the input carrier frequency estimated output by pre-filter 1 can be expected to remain relatively stable and accurate. In other words, instead of being given by Equation (17), the local carrier frequency $u_{k}^{carr}$ is calculated from:

(36) $$u_{k}^{carr}=\frac{\Delta {\hat{\theta }}_{k}^{carr}}{2\pi T_{coh}}+{\left\{-\frac{f_{L1}}{c}\left[e_{1}\left(v_{x}^{s}-v_{x}\right)+e_{2}\left(v_{y}^{s}-v_{y}\right)+e_{3}\left(v_{z}^{s}-v_{z}\right)\right]+f_{IF}\right\}|}_{k}+\frac{1}{2}{\hat{\dot{f}}}_{k}^{carr}T_{coh}$$

where $v_{x}$, $v_{y}$, $v_{z}$ are the corrected vehicle velocity components in ECEF coordinate system, $v_{x}^{s}$, $v_{y}^{s}$, $v_{z}^{s}$ are the satellite velocity components. Meanwhile, because vehicle dynamics have less effect on the code loop, generation of local code is still based upon the state feedback value $u^{code}$ given by Equation (26), namely, the structure of the code loop is unchanged.

## 4. Results and Discussion

This paper carried out static field tests and semi-physical simulations based on a telemetered missile trajectory to verify the validity of the proposed methods. In the static field test, the integration of GPS and INS is not involved, and only GPS is utilized to compare the performance of the baseband signal preprocessing models based on signal-filter and dual-filter structures. In the simulation, the navigation results of the ultra-tight GPS/INS integration are analyzed, and special attention is given to the further improvement of carrier frequency tracking accuracy.

### 4.1. Static Field Test

**Figure 3:**
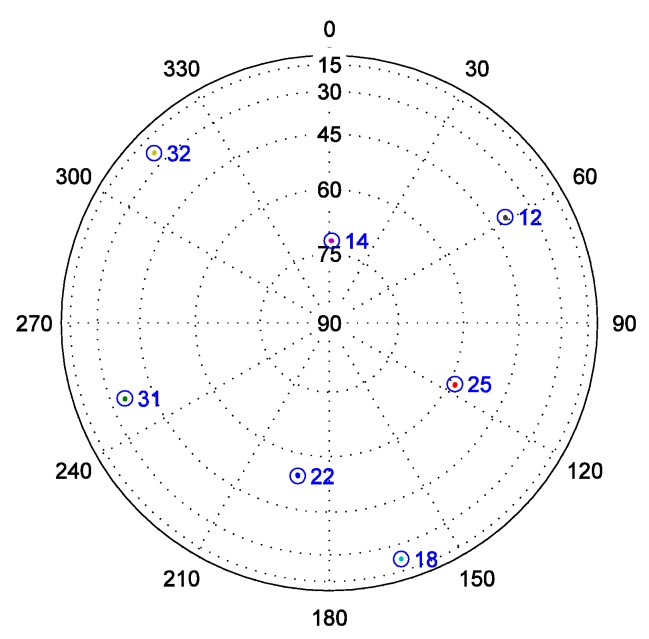
Satellites sky plot of the field test.

Although the baseband signal preprocessing model and the ultra-tight GPS/INS integration can both restrain noise better, the field test is carried out in an open-sky area ($N:39^{{}^{\circ}}57^{'}36.16^{"}$, $E:116^{{}^{\circ}}19^{'}1.58^{"}$, Hgt:32.9m) and does not involve the weak signal problem. After collecting signals with a radio frequency front-end, seven satellites are chosen according to their high Carrier-to-Noise Ratio $\left(C/N_{0}\right)$ listed in Table 1. The sky plot of these satellites is shown in Figure 3, and the elevation of each satellite is always greater than $10^{{}^{\circ}}$ during the test.

**Figure 4:**
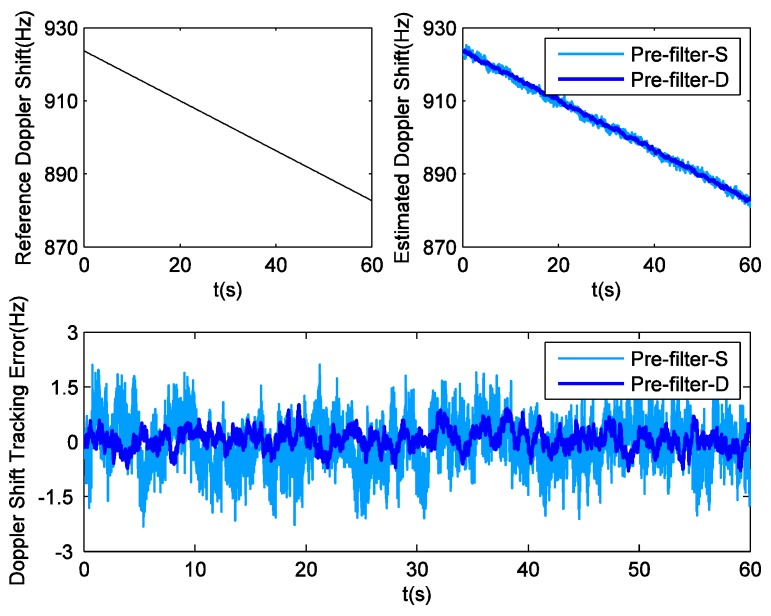
Doppler shift tracking error for PRN25 in field test.

**Figure 5:**
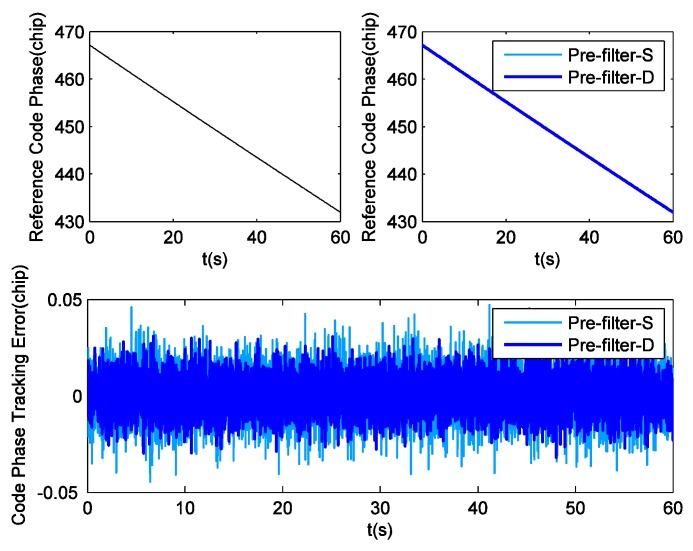
Code phase tracking error for PRN25 in field test.

Since weak signals are beyond the scope of this contribution, there is no need to employ INS to aid GPS tracking in static field tests. Hence this part just compares the performance of GPS receivers equipped with single pre-filter (pre-filter-S) and double pre-filters (pre-filter-D). First of all, in the tracking domain, the reference Doppler shifts can be provided by cubic spline fit interpolation. Then, taking PRN25 for example, Figure 4 shows the difference between Doppler shift tracking errors obtained by these two aforesaid methods. According to the statistical analysis, the Root Mean Square (RMS) value of the Doppler shift tracking errors corresponding to pre-filter-S is 0.487 Hz. For pre-filter-D, this value is 0.198 Hz. Obviously, pre-filter-S has a bad Doppler shift estimation outcome due to the influence of code phase error, while pre-filter-D achieves a significantly higher degree of precision. The decoupling between carrier tracking and code tracking proposes a 59.3 percent reduction in Doppler shift tracking error. Staying with the PRN25 example, Figure 5 shows the difference between the code phase tracking errors obtained by these two methods. It is observed that the new model proposed in this paper has slightly better performance. Specifically, the RMS values of code phase tracking errors corresponding to pre-filter-S and pre-filter-D are respectively 1.55$\;\times 10^{-2}$ chip and 1.43$\;\times 10^{-2}$ chip. The decoupling reduces the code phase tracking error by 7.7 percent.

**Figure 6:**
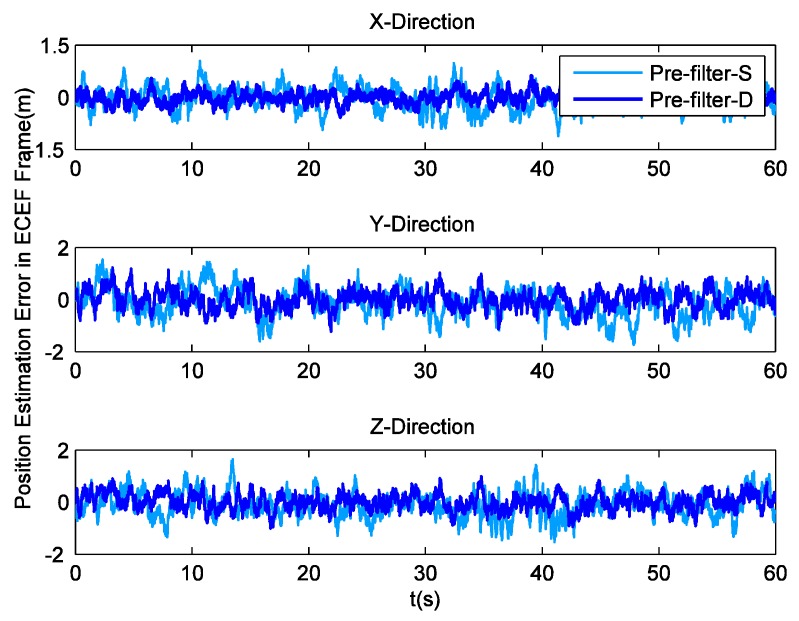
Position estimation errors with field data.

**Figure 7:**
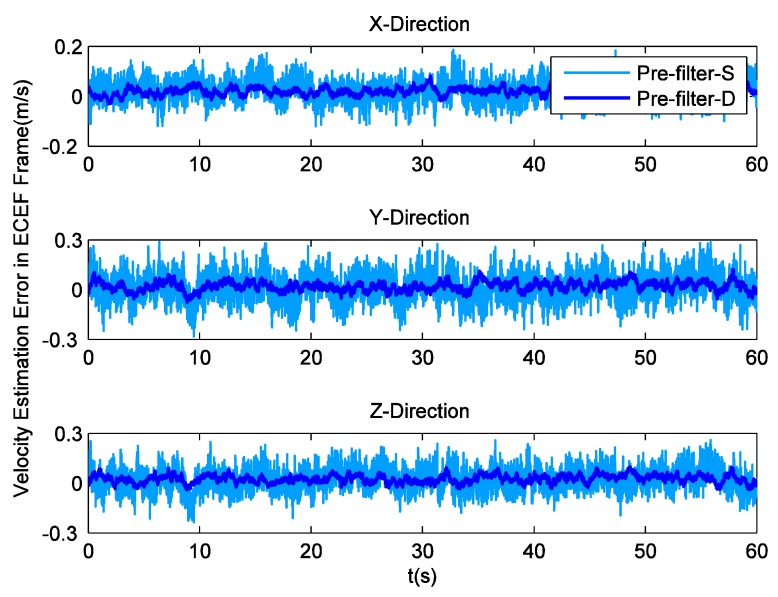
Velocity estimation errors with field data.

To analyze the impact of tracking parameters on position and velocity detection, receiver implements navigation through Least Squares (LS). The position estimation errors in the ECEF coordinate system are shown in Figure 6, while their corresponding RMS values are listed in Table 2. It can be seen that pre-filter-D makes positioning more precise. Then, Figure 7 illustrates the velocity estimation errors in ECEF frame, and Table 3 lists their RMS values. Obviously, there are multiple glitch impulses in the velocity estimation result output by the receiver employing pre-filter-S, which is quite similar to the situation in Doppler shift tracking. Sometimes the velocity errors in the y and z direction are even close to 0.3 m/s. On the contrary, the other receiver achieves comparatively stable and accurate outcomes. The absolute maximum values of the velocity errors are smaller than 0.1, 0.13 and 0.12 m/s, respectively.

**Figure 8:**
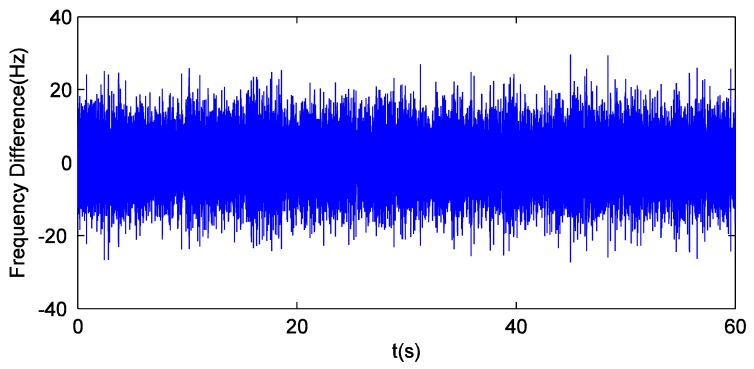
Frequency difference between local and input carriers in field tests.

It can be drawn from the analysis above that, using the new preprocessing model based on dual-filter makes GPS receiver behave better in the static field tests. Furthermore, it is worth mentioning that the frequency difference between input and local carrier is shown in Figure 8. Although the GPS tracking channel generates a local carrier with a high fluctuation from the input carrier, it can be interpreted as resulting from the design principle that $\Delta \theta_{k+1}^{carr}$ must return to 0 in the shortest amount of time. The analysis of Figure 4 indicates the precision of Doppler shift tracking is not reduced, that is, pre-filter 1 is insensitive to the wide fluctuation of local carrier frequency.

### 4.2. Semi-Physical Simulation Based on Telemetered Missile Trajectory

**Figure 9:**
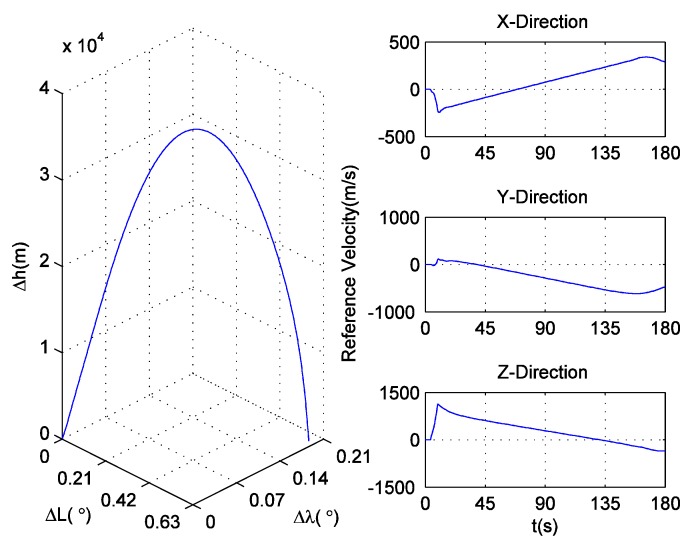
The reference trajectory for simulation.

**Figure 10:**
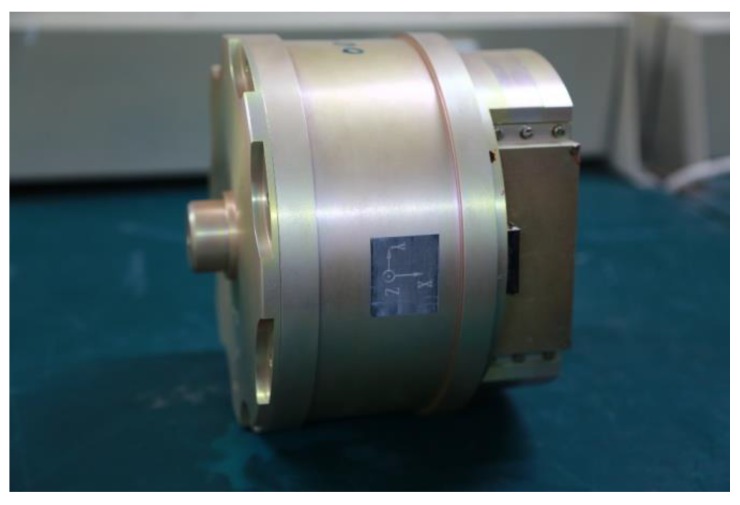
The (strapdown) INS used in the missile test.

**Figure 11:**
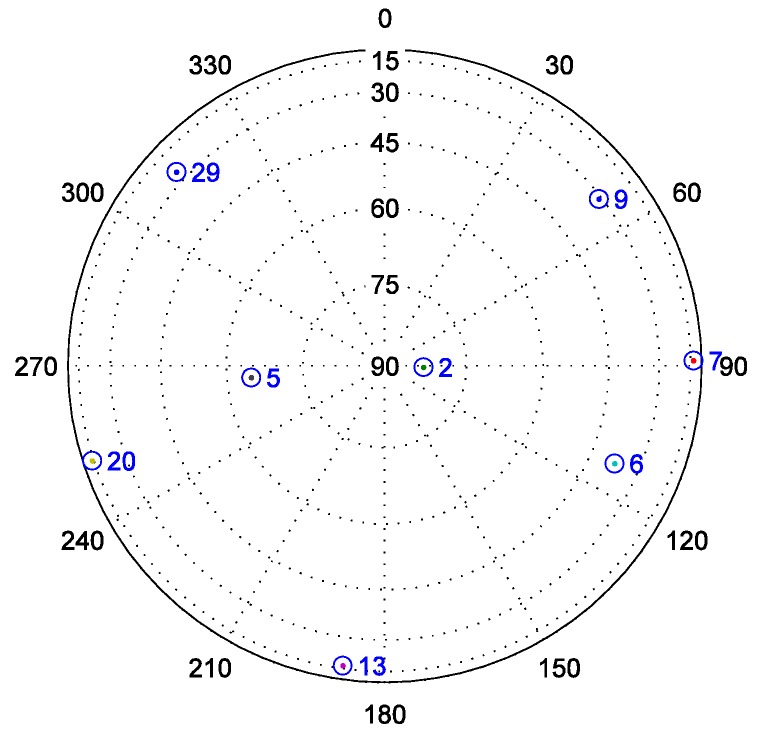
Satellites sky plot of the simulation.

Highly dynamic conditions, which are needed for validating the effectiveness of the ultra-tight GPS/INS integration, are extremely difficult to achieve in practice. To simulate this condition, in this paper, a missile trajectory lasting 180.23 s is derived from Fiber Optic Gyroscope (FOG) and accelerometer data, which are telemetered during a missile test, and is treated as the reference trajectory. The position of the missile relative to the launch site in geodetic coordinate system, and the velocity of the missile in ECEF coordinate system are shown in Figure 9. Figure 10 shows the (strapdown) INS used in the missile test. The statistical characteristics of the FOG and accelerometer are listed in Table 4. If the same errors in turn are added to the original FOG and accelerometer data, a set of new data can be generated. The trajectory corresponding to these new inertial measurements can be used as the INS navigation result, which needs to be calibrated by the ultra-tight GPS/INS integration. In addition, based on the reference trajectory as well as the satellite configuration at the missile launch site shown in Figure 11, the IF signal can be simulated.

**Figure 12:**
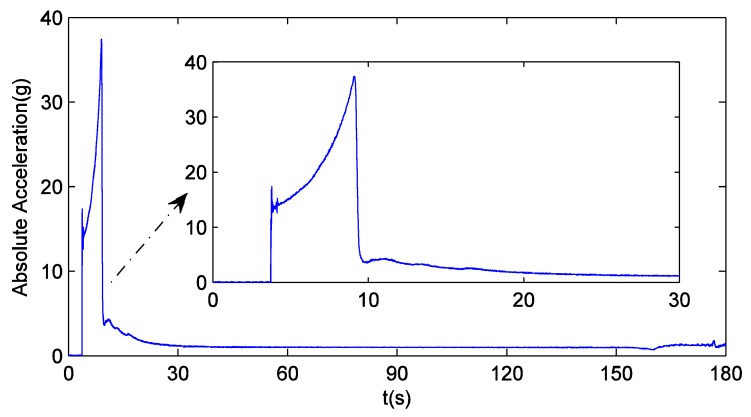
The absolute acceleration of the missile.

**Figure 13:**
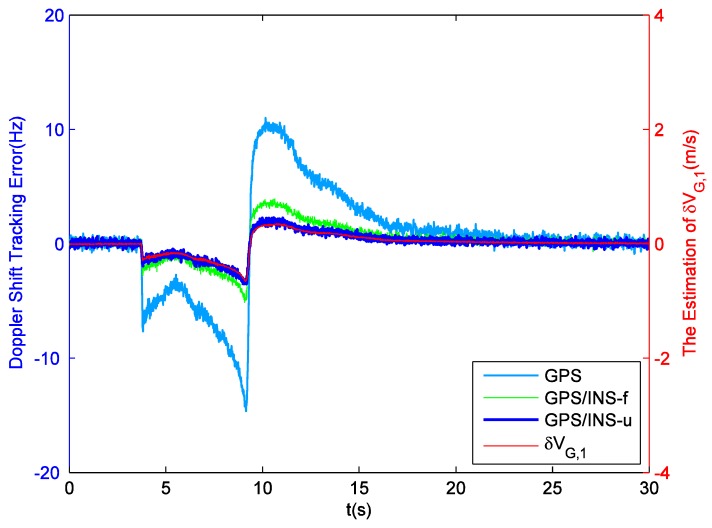
Doppler shift tracking error for PRN5 and the estimation of $\delta v_{G,1}$.

Figure 12 provides a close look at the absolute acceleration of the missile following the reference trajectory. It is observed that this value does not remain unchanged during the missile ascent phase. Indeed, this value has abrupt changes at about 4 s and 9 s. That usually means that even baseband signal preprocessing cannot ensure the carrier frequency estimation accuracy. Taking PRN5 as example, the Doppler shift tracking error obtained from the independent GPS receiver equipped with pre-filter-D is shown in Figure 13. Obviously, the rapid variation of acceleration introduces a significant error into the estimated Doppler shift after 4 s, and the sudden reversal of jerk at 9 s causes the sign of this error to change. As a comparison, the same dynamic simulation is also performed with the ultra-tight GPS/INS integration. The local carrier generation can be directly controlled by the frequency derived from the external navigation information (GPS/INS-f), but even so, the carrier tracking loop is still equivalent to a third-order phase locked loop. Hence, in the integrated system, the Doppler shift tracking error follows the same trend as the absolute acceleration. However, the absolute maximum value of this error drops to 5 Hz from 15 Hz, and the whole tracking process goes more smoothly. According to Equation (36), the external navigation information can also be used to correct the state feedback value. In this case, local carrier generation is controlled by state feedback (GPS/INS-u), and the absolute maximum value of the Doppler shift estimation error is 3 Hz. The following discussions focus on GPS/INS-u. It can be seen from Equation (29) that $\delta v_{G}$ is proportional to the estimation error of input carrier frequency, so Figure 13 also shows that the estimation of $\delta v_{G,1}$ (PRN5 is locked by channel #1) is accurate. In this sense, the proposed navigation filter can ensure INS is properly calibrated. If the traditional 17-dimensional filter is applied, in fact, the significant Doppler shift tracking error caused by missile ascent will lead the navigation results to gradually deviate from the reference trajectory until the whole integrated system cannot work.

**Figure 14:**
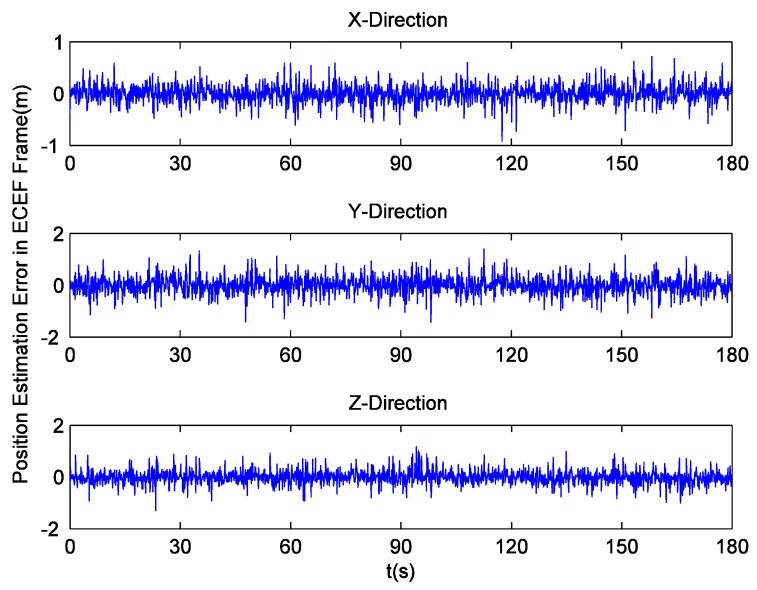
GPS position estimation errors with simulation data.

**Figure 15:**
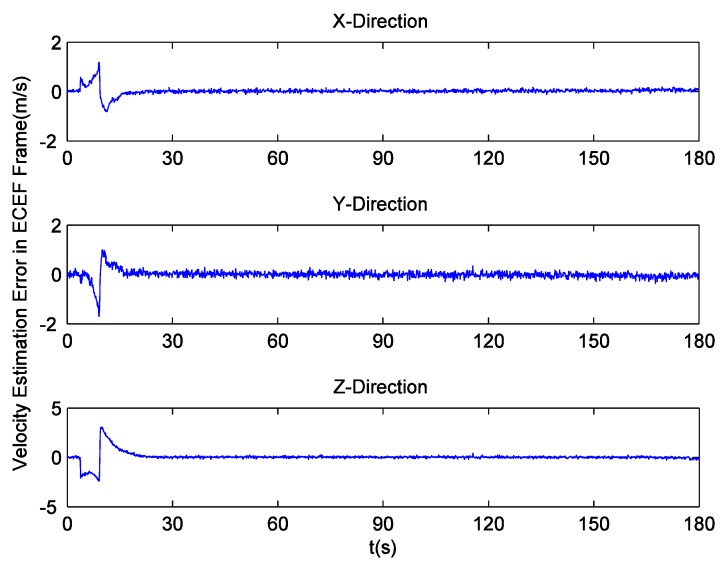
GPS velocity estimation errors with simulation data.

**Figure 16:**
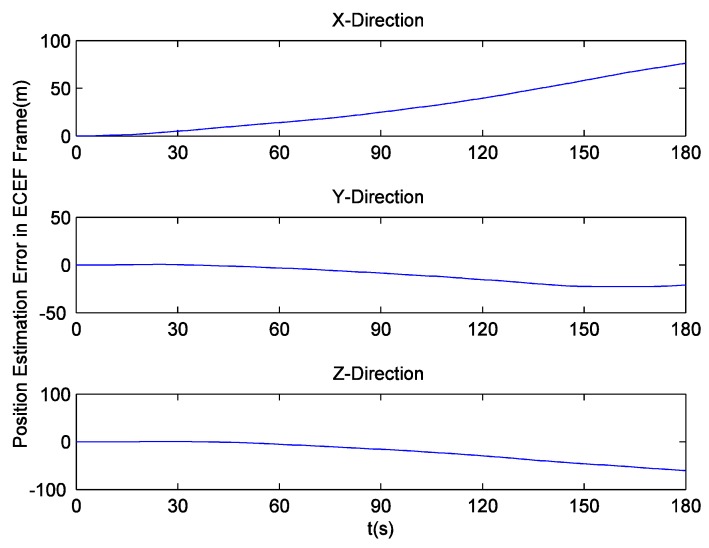
INS position estimation errors.

**Figure 17:**
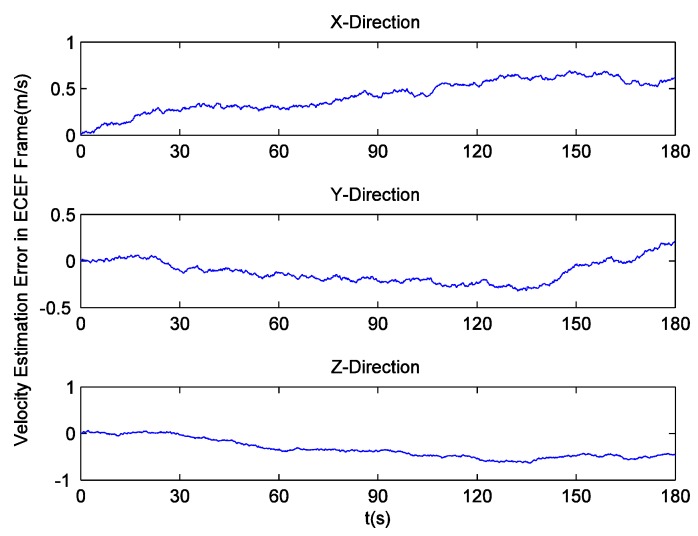
INS velocity estimation errors.

**Figure 18:**
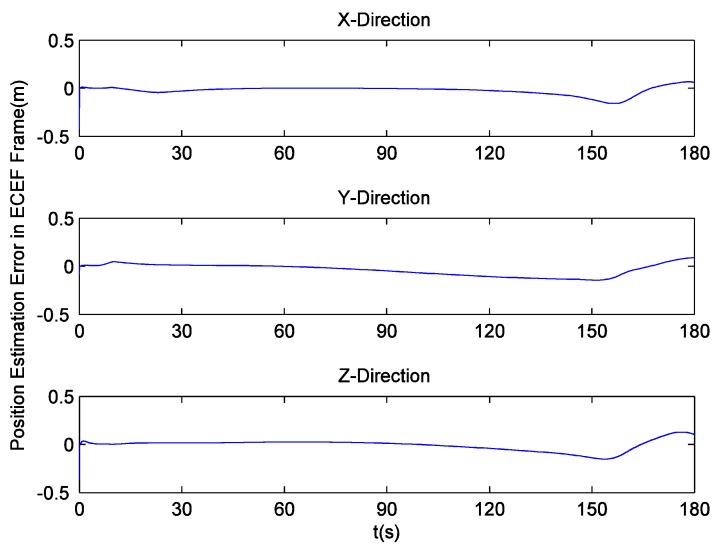
GPS/INS position estimation errors.

**Figure 19:**
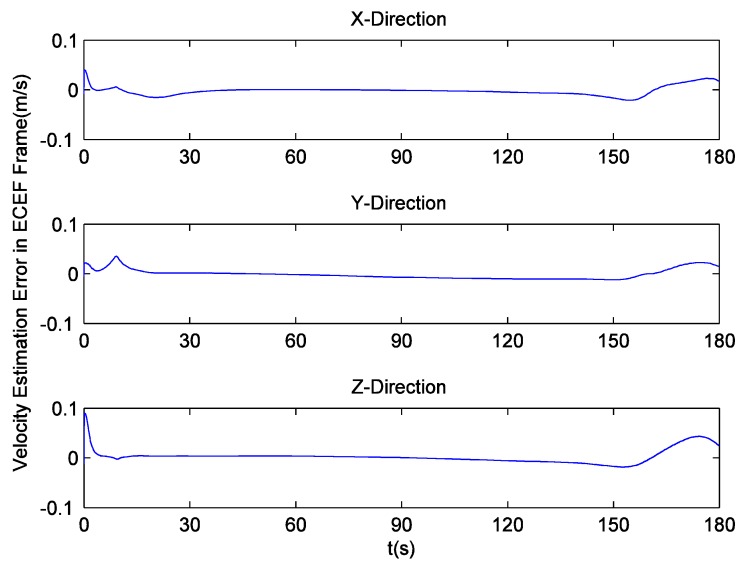
GPS/INS velocity estimation errors.

Since there is no loss of lock in dynamic simulation, the independent GPS receiver equipped with the new preprocessing model can implement navigation. However, in navigation domain, if KF is applied, the large error contained in the estimated Doppler shift may lead to inaccurate positioning results. Hence the independent GPS receiver still uses LS to obtain navigation results. Figure 14 and Figure 15 respectively show its position and velocity estimation errors in ECEF coordinate system. And Figure 16 and Figure 17 show the INS navigation errors in the same coordinate system. Meanwhile, navigation errors of the ultra-tight GPS/INS integration, namely the difference between the corrected inertial navigation information and reference values are shown in Figure 18 and Figure 19. Obviously, the velocity components estimated by the independent GPS receiver have large errors, which are on the order of 3 m/s, during the missile ascent phase. This is consistent with the carrier tracking result. For the case of INS, there is no sudden change in navigation errors. That is because INS is robust to vehicle dynamics. However, INS has the problem of error accumulation. At 180 s, the position errors are 76.3, −21.1 and −60.4 m, while the velocity errors are 0.6, 0.2 and −0.5 m/s. On the other hand, employing the navigation filter with expanded dimension, the ultra-tight GPS/INS integration can provide more accurate navigation results. The absolute maximum values of position errors and velocity errors are smaller than 0.5 m and 0.1 m/s, respectively.

**Figure 20:**
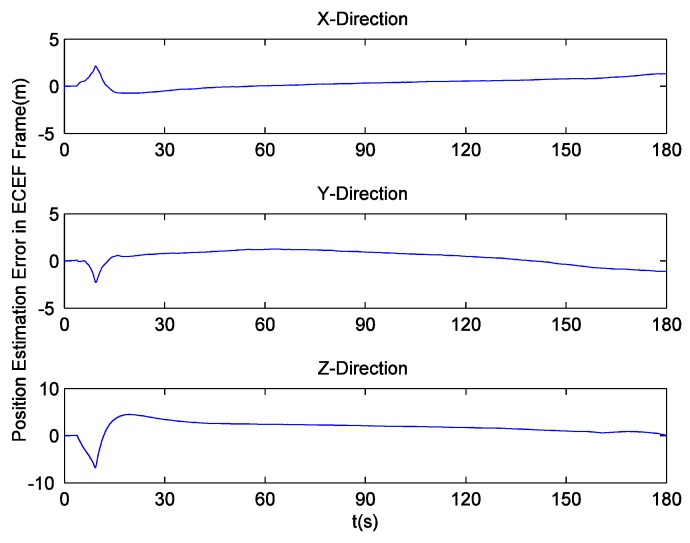
GPS/INS (tight integration, traditional navigation filter) position estimation errors.

**Figure 21:**
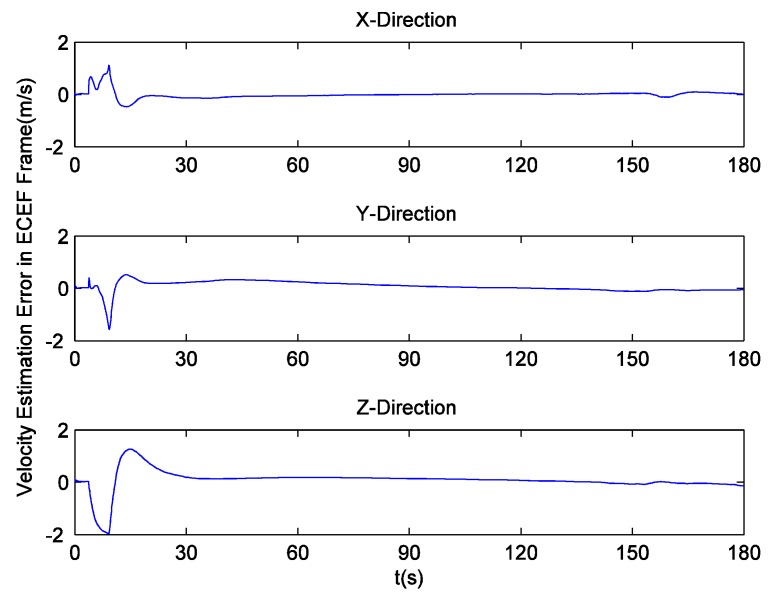
GPS/INS (tight integration, traditional navigation filter) velocity estimation errors.

As mentioned before, the traditional navigation filter cannot maintain the estimation accuracy of INS errors when the GPS receiver operates under harsh conditions. Once the incorrect navigation information is utilized to generate local carrier frequency, the Doppler shift tracking error will further increase, so it can be concluded that the proposed navigation filter elegantly avoids this vicious circle. Here, a tight GPS/INS integration based on the traditional navigation filter is realized to show the harmful effect of carrier tracking errors in detail. The corresponding navigation errors are shown in Figure 20 and Figure 21. It can be observed that the tight integration gradually achieves acceptable navigation accuracy as time goes on, but during the missile ascent phase, the velocity errors of this integration are on the order of 2 m/s, and are much larger than those of INS.

## 5. Conclusions

This paper proposes a baseband signal preprocessing model, which has two pre-filters and uses state feedback values to control the generation of local carrier and code. Compared with the conventional approach based on a single-filter structure, this model limits the impact of code phase error on carrier tracking, and hence enables the receiver to achieve better navigation precision. Furthermore, this model can ensure the stationarity of the estimation of input carrier frequency, even in the case of local carrier frequencies with wide fluctuation. In fact, compared with a popular error-state pre-filter, even pre-filter-S proposed in this paper performs better in the tracking domain due to the benefits of state feedback.

To further promote the tracking and navigation performance, a specific ultra-tight GPS/INS integration is designed on the basis of the new preprocessing model. Since the pseudorange rate estimation error in each channel has been treated as the additional state variable, the proposed navigation filter ensures the estimation of INS errors will not be contaminated. Moreover, in this integration, although the corrected inertial navigation information is used to estimate the carrier frequency, the tracking channel still aims to make the phase error return to 0 in the shortest amount of time. The results of the semi-physical simulation based on a telemetered missile trajectory indicate that navigation solutions with higher accuracy can be achieved. Meanwhile, because tracking is assisted by external navigation information, the error contained in the estimated Doppler shift can be reduced substantially.

## Figures and Tables

**Table 1:** $C/N_{0}$ of selected satellites.

Satellite	$C/N_{0}$(dB·Hz)	Satellite	$C/N_{0}$(dB⋅Hz)
PRN22	48.881	PRN14	45.612
PRN31	48.025	PRN32	45.693
PRN25	45.987	PRN12	45.202
PRN18	48.086		

**Table 2:** RMS of position estimation errors.

Method	RMS of Position Error (m)		
	X	Y	Z
Pre-filter-S	0.359	0.585	0.519
Pre-filter-D	0.171	0.367	0.315

**Table 3:** RMS of velocity estimation errors.

Method	RMS of Velocity Error (m/s)		
	X	Y	Z
Pre-filter-S	0.044	0.085	0.072
Pre-filter-D	0.026	0.036	0.036

**Table 4:** Gyro and accelerometer specifications.

	Gyros	Accelerometers
In-Run Bias Error	0.5°/h	2$\;\times 10^{-4}\;$g
Noise (1σ, smoothing window: 10 ms)	15°/h	2$\;\times 10^{-2}\;$g

**Figure B1:**
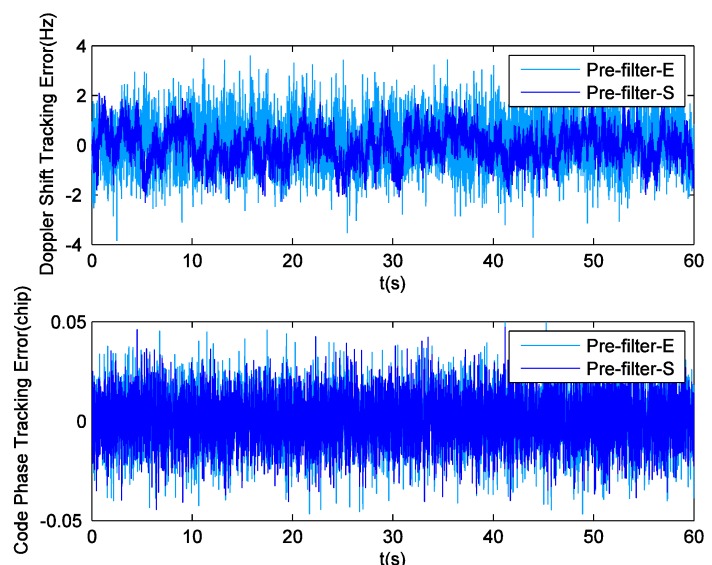
Tracking errors of pre-filter-E and pre-filter-S.

## Appendix A

First, the items in Equation (1) are numbered as follows:

(A1) $$\Delta \theta_{k}^{carr}=\Delta \theta_{k-1}^{carr}+2\pi f_{k-1}^{carr}T_{coh}+\pi {\dot{f}}_{k-1}^{carr}T_{coh}^{2}-2\pi u_{k-1}^{carr}T_{coh}$$

(A2) $$f_{k}^{carr}=f_{k-1}^{carr}+{\dot{f}}_{k-1}^{carr}T_{coh}$$

(A3) $${\dot{f}}_{k}^{carr}={\dot{f}}_{k-1}^{carr}$$

Obviously, Equation (A3) can be rewritten as:

(A4) $${\dot{f}}_{k-1}^{carr}={\dot{f}}_{k}^{carr}$$

Substitution of Equation (A4) into Equation (A2) results in:

$$f_{k}^{carr}=f_{k-1}^{carr}+{\dot{f}}_{k}^{carr}T_{coh}$$

then:

(A5) $$f_{k-1}^{carr}=f_{k}^{carr}-{\dot{f}}_{k}^{carr}T_{coh}$$

and substitution of Equations (A4) and (A5) into Equation (A1) results in:

$$\begin{array}{ll} \Delta \theta_{k}^{carr} & =\Delta \theta_{k-1}^{carr}+2\pi \left(f_{k}^{carr}-{\dot{f}}_{k}^{carr}T_{coh}\right)T_{coh}+\pi {\dot{f}}_{k}^{carr}T_{coh}^{2}-2\pi u_{k-1}^{carr}T_{coh}\\ & =\Delta \theta_{k-1}^{carr}+2\pi f_{k}^{carr}T_{coh}-\pi {\dot{f}}_{k}^{carr}T_{coh}^{2}-2\pi u_{k-1}^{carr}T_{coh} \end{array}$$

then:

(A6) $$\Delta \theta_{k-1}^{carr}=\Delta \theta_{k}^{carr}-2\pi f_{k}^{carr}T_{coh}+\pi {\dot{f}}_{k}^{carr}T_{coh}^{2}+2\pi u_{k-1}^{carr}T_{coh}$$

Finally, Equation (3) can be obtained by combining Equations (A4)–(A6) together.

## Appendix B

The filter, whose state vector is ${\left[\Delta \theta_{k}^{carr}\;\Delta f_{k}^{carr}\;\Delta {\dot{f}}_{k}^{carr}\;\Delta \theta_{k}^{code}\right]}^{T}$, is a popular baseband signal preprocessing model. Herein, $\Delta f_{k}^{carr}=f_{k}^{carr}-u_{k}^{carr}$, $\Delta {\dot{f}}_{k}^{carr}={\dot{f}}_{k}^{carr}-0$. To focus on analyzing the benefits of decoupling between carrier tracking and code tracking, this filer should be further improved. First, consider the equations derived from Equation (18):

(B1) $$f^{code}=\beta f^{carr}+\beta \left(f_{L1}-f_{IF}\right)$$

(B1) $${\dot{f}}^{code}=\beta {\dot{f}}^{carr}$$

where $\beta =\frac{f_{CA}}{f_{L1}}$. The new state variables $\Delta \theta_{k}^{carr}$, $f_{k}^{carr}$, ${\dot{f}}_{k}^{carr}$ and $\Delta \theta_{k}^{code}$ have the following recurrence relations:

(B3) $$\left\{ \begin{array}{l} \Delta \theta_{k}^{carr}=\Delta \theta_{k-1}^{carr}+2\pi f_{k-1}^{carr}T_{coh}+\pi {\dot{f}}_{k-1}^{carr}T_{coh}^{2}-2\pi u_{k-1}^{carr}T_{coh}\\ f_{k}^{carr}=f_{k-1}^{carr}+{\dot{f}}_{k-1}^{carr}T_{coh}\\ {\dot{f}}_{k}^{carr}={\dot{f}}_{k-1}^{carr}\\ \Delta \theta_{k}^{code}=\Delta \theta_{k-1}^{code}+\beta f_{k-1}^{carr}T_{coh}+\frac{1}{2}\beta {\dot{f}}_{k-1}^{carr}T_{coh}^{2}-u_{k-1}^{code}T_{coh}+\beta \left(f_{L1}-f_{IF}\right)T_{coh} \end{array}\right.$$

The relation between the observation $\Delta \theta_{k,k-1}^{carr}$ and state variables can still be represented by Equation (4), while $\Delta \theta_{k,k-1}^{code}$ is written as:

(B4) $$\Delta \theta_{k,k-1}^{code}=\Delta \theta_{k}^{code}-\frac{1}{2}\beta f_{k}^{carr}T_{coh}+\frac{1}{6}\beta {\dot{f}}_{k}^{carr}T_{coh}^{2}+\frac{1}{2}u_{k-1}^{code}T_{coh}-\frac{1}{2}\beta \left(f_{L1}-f_{IF}\right)T_{coh}$$

Then, the state equation and observation equation of pre-filter-S can be expressed as:

(B5) $$\left\{ \begin{array}{l} X_{k}^{caco}=\Phi_{k,k-1}^{caco}X_{k-1}^{caco}+M_{k-1}^{caco}+W_{k-1}^{caco}\\ Z_{k}^{caco}=H_{k}^{caco}X_{k}^{caco}+N_{k}^{caco}+V_{k}^{caco} \end{array}\right.$$

where:

(B6) $$\Phi_{k,k-1}^{caco}=\left[\begin{array}{cccc} 1 & 2\pi T_{coh} & \pi T_{coh}^{2} & 0\\ 0 & 1 & T_{coh} & 0\\ 0 & 0 & 1 & 0\\ 0 & \beta T_{coh} & \frac{1}{2}\beta T_{coh}^{2} & 1 \end{array}\right]$$

(B7) $$M_{k-1}^{caco}={\left[\begin{array}{cccc} -2\pi T_{coh}u_{k-1}^{carr} & 0 & 0 & \beta \left(f_{L1}-f_{IF}\right)T_{coh}-T_{coh}u_{k-1}^{code} \end{array}\right]}^{T}$$

(B8) $$H_{k}^{caco}=\left[\begin{array}{cccc} 1 & -\pi T_{coh} & \frac{1}{3}\pi T_{coh}^{2} & 0\\ 0 & -\frac{1}{2}\beta T_{coh} & \frac{1}{6}\beta T_{coh}^{2} & 1 \end{array}\right]$$

(B9) $$N_{k}^{caco}={\left[\begin{array}{cc} \pi T_{coh}u_{k-1}^{carr} & -\frac{1}{2}\beta \left(f_{L1}-f_{IF}\right)T_{coh}+\frac{1}{2}T_{coh}u_{k-1}^{code} \end{array}\right]}^{T}$$

(B10) $$Z_{k}^{caco}=\left[\begin{array}{c} \Delta \theta_{k,k-1}^{carr}\\ \Delta \theta_{k,k-1}^{code} \end{array}\right]$$

while the feedback for carrier tracking is still represented by Equation (17), the feedback for code tracking is expressed as:

(B11) $$u_{k}^{code}=\frac{1}{T_{coh}}\Delta {\hat{\theta }}_{k}^{code}+\beta {\hat{f}}_{k}^{carr}+\frac{1}{2}\beta {\hat{\dot{f}}}_{k}^{carr}T_{coh}+\beta \left(f_{L1}-f_{IF}\right)$$

Furthermore, it is noted that $\Delta \theta_{k,k-1}^{code}$ is uncorrelated with $\Delta \theta_{k,k-1}^{carr}$ [15]. In fact, based on statistical analysis, it can be drawn that the correlation coefficient between $\Delta \theta_{k,k-1}^{carr}$ and $\Delta \theta_{k,k-1}^{code}$ is usually smaller than 0.01. The measurement covariance matrix $R_{k}^{caco}$, therefore, is set as:

(B12) $$R_{k}^{caco}=\left[\begin{array}{cc} \sigma_{1}^{2} & 0\\ 0 & \sigma_{2}^{2} \end{array}\right]$$

where $\sigma_{1}^{2}$ and $\sigma_{2}^{2}$ are the variances at the outputs of the carrier and code discriminators respectively. In general, $\sigma_{1}^{2}$ and $\sigma_{2}^{2}$ can be calculated as follows [10]:

(B13) $$\sigma_{1}^{2}=\frac{1}{2C/N_{0}T_{coh}}\left(1+\frac{1}{2C/N_{0}T_{coh}}\right)$$

(B14) $$\sigma_{2}^{2}=\frac{d}{4C/N_{0}T_{coh}}\left[1+\frac{2}{\left(2-d\right)C/N_{0}T_{coh}}\right]$$

where d is the early-late correlator spacing.

Pre-filter-S usually has better performance than the popular error-state filter (pre-filter-E). Taking PRN25 for example, Figure B1 shows the differences between tracking errors obtained by pre-filter-E and pre-filter-S. According to statistic analysis, the RMS values of Doppler shift and code phase tracking errors corresponding to pre-filter-E are respectively 1.028 Hz and 1.62$\;\times 10^{-2}$ chip. For pre-filter-S, these two values are 0.487 Hz and 1.55$\;\times 10^{-2}$ chip.
